# Sensing Trace-Level Metal Elements in Water Using Chirped Femtosecond Laser Pulses in the Filamentation Regime

**DOI:** 10.3390/s22228775

**Published:** 2022-11-13

**Authors:** Shanming Chen, Xun Cong, Junyan Chen, Hongwei Zang, Helong Li, Huailiang Xu

**Affiliations:** 1 State Key Laboratory of Integrated Optoelectronics, College of Electronic Science and Engineering, Jilin University, Changchun 130012, China; 2 Institute of Atomic and Molecular Physics, Jilin University, Changchun 130012, China; 3 State Key Laboratory of Precision Spectroscopy & Chongqing Institute, East China Normal University, Shanghai 200062, China

**Keywords:** femtosecond laser pulse, filament, water, limit of detection, quantitative analysis

## Abstract

Femtosecond filament-induced breakdown spectroscopy (FIBS) is an efficient approach in remote and in situ detection of a variety of trace elements, but it was recently discovered that the FIBS of water is strongly dependent on the large-bandgap semiconductor property of water, making the FIBS signals sensitive to laser ionization mechanisms. Here, we show that the sensitivity of the FIBS technique in monitoring metal elements in water can be efficiently improved by using chirped femtosecond laser pulses, but an asymmetric enhancement of the FIBS intensity is observed for the negatively and positively chirped pulses. We attribute the asymmetric enhancement to their different ionization rates of water, in which the energy of the photons participating in the ionization process in the front part of the negatively chirped pulse is higher than that in the positively chirped pulse. By optimizing the pulse chirp, we show that the limit of detection of the FIBS technique for metal elements in water, e.g., aluminum, can reach to the sub-ppm level, which is about one order of magnitude better than that by the transform-limited pulse. We further examine the FIBS spectra of several representative water samples including commercial mineral water, tap water, and lake water taken from two different environmental zones, i.e., a national park and a downtown business district (Changchun, China), from which remarkably different concentrations of Ca, Na, and K elements of these samples are obtained. Our results provide a possibility of using FIBS for direct and fast metal elemental analysis of water in different field environments.

## 1. Introduction

Laser-induced breakdown spectroscopy (LIBS) is an advanced analysis technique for rapid, on-line, multi-element detection of materials with little to no preparation [1,2,3,4,5]. The intense tightly focusing laser beam induces the breakdown on the material, generating high-temperature and dense plasma. As the plasma gradually cools, atomic components in the excited states can emit characteristic fluorescence emissions, which provide qualitative and quantitative elemental information of materials.

The LIBS technique has shown great applicability in a variety of fields such as industrial production [6,7], food detection [8,9], combustion diagnostics [10,11], and environmental and biological application [12,13,14,15]. Particularly, it has been extensively employed for monitoring water quality with rapid, high-sensitivity, and simultaneous measurements of multiple metal contaminates in water [16,17,18]. Moreover, possible secondary pollution can be avoided in the LIBS analysis process due to the absence of chemical reagents. However, the drawback of the short Rayleigh range in the tightly focusing scheme requires a stable air–water interface to reduce the LIBS intensity fluctuation, limiting its field applications in natural water bodies having surface movements due to the morphology and water waves.

Recently, it was shown that femtosecond filament-induced breakdown spectroscopy (FIBS) could be a solution to overcome the impediment since the FIBS intensities were found to be insensitive to water waves [19]. This derives from the fact that the high constant laser intensity of about 50–100 TW/cm^2^ inside the filament can be sustained over several Rayleigh lengths or longer due to the dynamic balance between the self-focusing and self-generated plasma defocusing effects [20,21,22,23,24]. The high-intensity laser filament could be projected at a far distance, even in various complicated environments such as atmosphere and combustion flames, showing great potentials in remote sensing, fuel ignition, and laser fabrication [25,26,27]. These advantages of femtosecond laser filaments also make the technique of FIBS straightforwardly extendable to operation in the kilometer-scale range that cannot be achieved by LIBS with compact nanosecond laser systems, leading to the promising prospect of FIBS for remote applications even though femtosecond laser systems are costly and bulky. More recently, it was revealed that the large-bandgap semiconductor property of water plays a key role in FIBS of water, resulting in a strong dependence of the FIBS intensity on the input laser pulse duration and energy, which was ascribed to the tradeoff between the multiphoton ionization (MPI) and avalanche ionization (AI) effects [28].

In the present work, we experimentally investigate the pulse duration effect on FIBS, and observe an asymmetric enhancement of the FIBS intensity of the trace-level Al element in water, when the laser pulse is stretched along the negative and positive chirps. The FIBS intensity is optimized at around 200–300 fs, but the optimization intensity in the negative chirp case is much higher than that in the positive chirp case. Based on the semiconductor property of water, we ascribe the observed asymmetric dependence to the higher ionization rate of water induced by the negatively chirped pulse, leading to the stronger breakdown. With the optimization, we show that the limit of detection (LOD) of FIBS and its robustness against the water wave can be significantly improved as compared with those by the transform-limited pulse. We employ this technique to measure and evaluate the concentrations of metal elements such as Na, K, and Ca in different water samples including mineral water, tap water, and lake water, which shows the feasibility of the FIBS technique for monitoring trace metal pollution of water in different field environments.

## 2. Experimental Setup

**Figure 1:**
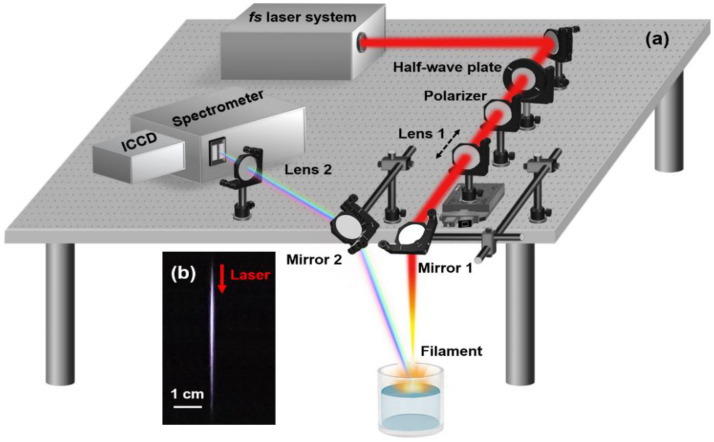
(**a**) Schematic diagram of experimental setup; (**b**) the filament image captured with a digital camera; the red arrow indicates the laser propagation direction.

Figure 1 illustrates the schematic experimental setup. Linearly polarized laser pulses with 800 nm central wavelength, 3.0 mJ pulse energy, and 500 Hz repetition rate were generated from a Ti:sapphire laser amplifier system (Spectra Physics, Spitfire ACE). The negative and positive laser chirps were introduced by the compressor of the laser system and the corresponding pulse durations were monitored by an autocorrelator (Spectra Physics, PSCOUNT2-NIR-PD). The laser pulses were loosely focused by a fused silica lens (Lens 1, focus length *f* = 100 cm) and then reflected by a mirror (Mirror 1) with high reflectivity at around 800 nm to propagate vertically. The focused laser formed a single filament with a diameter of about 100 μm, which interacted with the surface of water samples in a large column container to generate a plasma plume. The pulse energy was controlled by a half-wave plate and a polarizer, in which the transmission portion of the laser energy through the polarizer can be adjusted by rotating the half wave plate. Since the pulse duration can strongly affect the critical power of the input laser pulse for the Kerr self-focusing, both the filament length and size change as the laser pulse is chirped. Therefore, Lens 1 was fixed on a one-dimensional moving stage to carefully adjust the focus-to-surface distance (FSD), optimizing the plasma emission intensity [28].

The plasma emissions from the water surface were collected in the backward direction by an aluminum-coated mirror (Mirror 2) with a diameter of 50.8 mm. In this collection system, the distance between Mirror 2 and the water surface is about 1 m, so that the signal is regarded to be emitted from a point source. The signal was then focused by a fused silica lens (Lens 2, *f* = 6 cm) onto the entrance slit of the spectrometer (Andor Shamrock SR-500i) equipped with an intensified charge coupled device camera (ICCD, Andor iStar, Belfast, England). The input plasma signal was dispersed by a 1200 grooves/mm grating and then recorded by an ICCD camera. In the measurements, the entrance slit was set to be 200 μm and the ICCD gate delay and window were set to be 200 and 1000 ns, respectively. With these settings, the plasma continuum background can be well reduced. The data for each spectrum were accumulated over 5000 laser shots.

## 3. Results and Discussion

**Figure 2:**
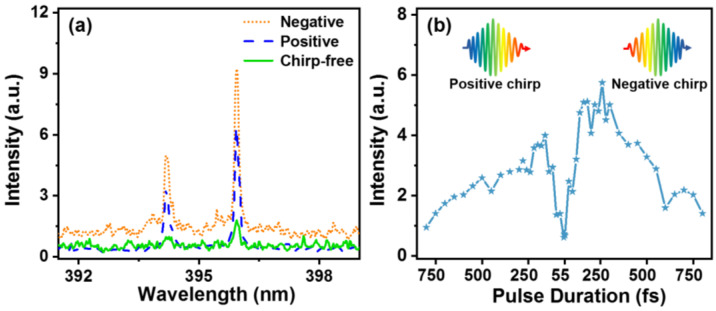
(**a**) FIBS emission spectra obtained respectively by a chirp-free 55 fs (solid), a negatively chirped 200 fs (dotted dash), and a positively chirped 200 fs (dash) pulse; (**b**) FIBS intensities of Al atoms at 396.2 nm obtained with different pulse durations under the negative chirp and positive chirp conditions.

Figure 2 shows the three FIBS spectra of a 1000 ppm Al water sample in the wavelength range from 390 to 402 nm obtained respectively by transform-limited 55 fs, positively chirped 200 fs, and negatively chirped 200 fs laser pulses. We employed Al as the sample because Al is one of the most representative metal pollutants. The laser energy was set to be 1.5 mJ. Two distinct spectral peaks at 394.4 and 396.2 nm can be clearly seen, which are assigned to the 3s^2^4s ^2^S_1/2_—3s^2^3p ^2^S_1/2_ and 3s^2^4s ^2^S_1/2_—3s^2^3p ^2^S_3/2_ transitions of the atomic Al, respectively. It can be observed that the intensities of the FIBS signals obtained in both the negatively and positively chirped 200 fs cases are much higher than that in the chirp-free 55 fs case. Interestingly, the signal intensities in the negatively chirped case are obviously stronger than those in the positively chirped case although the driven laser pulses have the same pulse duration of about 200 fs.

To clearly show the laser chirp effect on the FIBS intensity of water, we systematically investigate the dependence of the 396.2 nm emission intensity of Al atoms on the chirp of the pulse duration, as shown in Figure 2b. The signal fluctuation may originate from the instability of the filament length and position that are very sensitive to the laser energy fluctuation. It can be seen that the emission intensities in both the negative and positive chirp cases are first enhanced, stay constant, and then are decreased, which can be ascribed to the tradeoff between the MPI and AI effects in the breakdown of water induced by an intense femtosecond laser [28]. However, it can be seen that the FIBS intensity in the plateau region beginning at around 200 fs for the negatively chirped case is about 1.5 times stronger than that for the positively chirped case, exhibiting an asymmetric emission intensity enhancement effect, which has not been reported previously.

The physical mechanism underlying the asymmetric enhancement phenomenon is interpreted based on the ultrafast photoionization model of liquid water as follows. Since liquid water can be regarded as a 9 eV band-gap semiconductor material [29], six 800 nm photons (about 1.55 eV) are required to be simultaneously absorbed to excite the bound electrons on the valence band to the conduction band, generating the “seeding” electrons for the subsequent AI process, and thus the breakdown process of water by intense femtosecond laser excitation is highly sensitive to the MPI rate. It should be noted that MPI rate increases with the increasing photon frequency [30]. Since the higher optical frequencies lie at the pulse front part in the negatively chirped case, as illustrated in the insets of Figure 2b, more “free” electrons can be produced by the MPI effect, which is in favor of the following AI process, leading to a stronger FIBS intensity than that of the positively chirped pulse.

**Figure 3:**
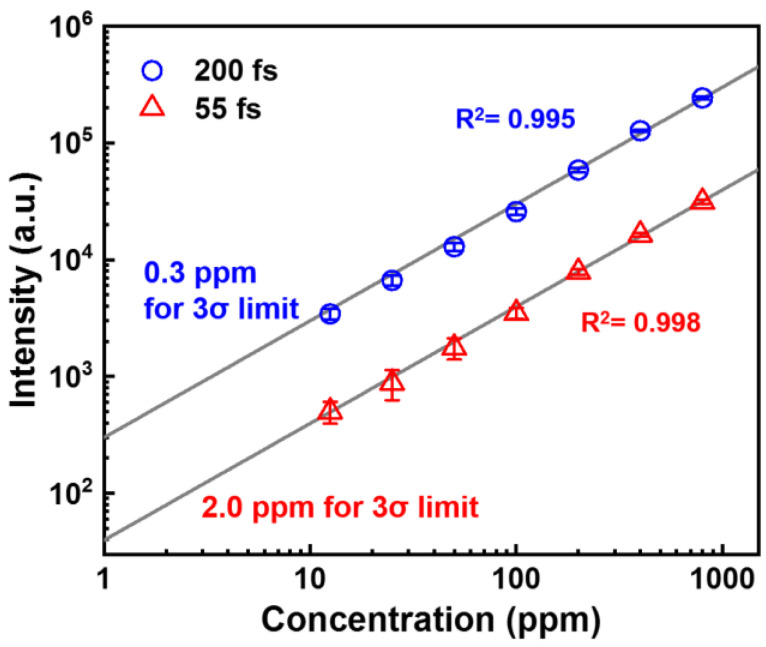
Dependences of the FIBS intensities on the Al concentrations for the chirp-free 55 fs (triangle) and negatively chirped 200 fs (circle) pulses.

Next, we investigate the effect of the laser chirp on the quantitative measurement ability of the FIBS technique by recording the FIBS spectra at different Al concentrations in water ranging from 12.5 to 800 ppm using the chirp-free 55 fs and negatively chirped 200 fs pulses. Shown in Figure 3 are the plots of the characteristic emission intensities of Al atoms at 396.2 nm as a function of the Al concentration. In the measurements, the pulse energy was set to be 3 mJ. The data can be fitted well by a linear function, showing that the FIBS intensity linearly increases with the increasing Al concentration. Based on the measurements, the LOD is calculated to be 0.3 ppm and 2.0 ppm, respectively, for chirped pulse and chirp-free cases, according to the definition of the International Union of Pure and Applied Chemistry, LOD = $3\sigma_{BG}S^{-1}$, where *σ_BG_* is the standard deviation of the signal and *S* is the slope of the calibration curve. Clearly, the obtained LOD results indicate that the introduction of the laser chirp can significantly improve the LOD of the filament-based LIBS technique. Since the signal was collected with a very simple geometry, it is possible to further improve the LOD by increasing the collection efficiency by the use of a large telescope system in a co-axial scheme.

**Figure 4:**
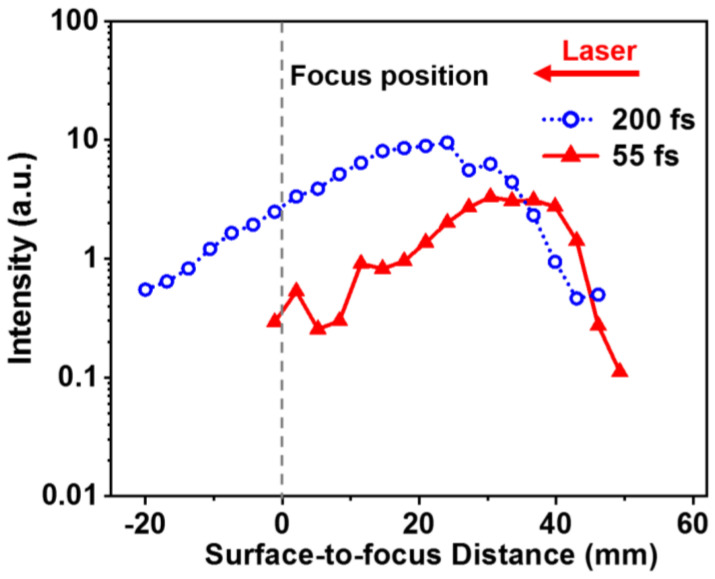
FIBS intensities obtained at different surface-to-focus distances by the excitations of the chirp-free 55 fs (triangle) and negatively chirped 200 fs (circle) pulses. The gray dashed line is the focal position of the lens.

Furthermore, we compare the dependences of the emission intensity of Al atoms at 396.2 nm on the FSD for the chirp-free 55 fs and the negatively chirped 200 fs pulses, as shown in Figure 4. It can be seen that the both the FIBS intensities are insensitive to the variation of the FSD, which is due to the constant clamped laser intensity inside the long filament core. In addition, the FIBS intensity distribution is extended more along the filament to the focal position of the lens in the negatively chirped 200 fs case, demonstrating the stronger robustness against water waves, which may be ascribed to the stronger AI effect in the longer chirped pulse. This may be interpreted as follows. Since the clamping intensity inside the filament slightly decreases as the pulse duration increases, which leads to a reduced MPI effect, the stronger signal intensity in the chirped pulse thus implies that the AI effect plays a crucial role in the robustness enhancement due to the more AI cycles during the filament–water interaction.

**Figure 5:**
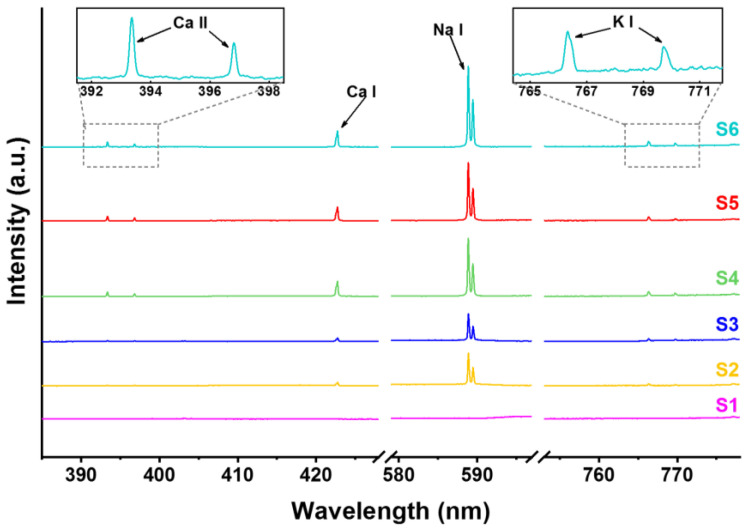
The negatively chirped FIBS spectra for six representative water samples: (S**1**) Watsons, (S**2**) Nongfu Spring, (S**3**) Quanyangquan, and (S**4**) tap water as well as the wild lake water from (S**5**) Jingyuetan National Forest Park and (S**6**) Nanhu Park in Changchun.

In order to examine the practical application potential of the FIBS technique, we employed the optimized pulse duration at about 200 fs, and measured the metal elements of several representative water samples, which are, respectively, commercial drinkable water including purified water from Watsons (S1), mineral water from Nongfu Spring (S2), and Quanyangquan (S3), and tap water (S4) as well as lake water taken from two different environments, i.e., Jingyuetan National Forest Park (S5) and Nanhu Park (S6), where Jingyuetan National Forest Park is located in the southeastern part of Changchun, covering an area of ~100 km^2^ with 96% forest coverage rate, while Nanhu Park is a typical city park located at the center of the downtown area having heavy traffic and many buildings. As shown in Figure 5, multiple spectral peaks can be seen in the FIBS spectra of Samples S2–S6, which can be respectively assigned to the 4p ^2^P^°^_3/2–_4s ^2^S_1/2_ and 4p ^2^P^°^_1/2–_4s ^2^S_1/2_ transitions of Ca II at around 393.4 and 396.9 nm, the 4s4p ^1^P^°^_1–_4s^2 1^S_0_ transition of Ca I at around 422.7 nm, the 3p ^2^P^°^_3/2–_3s ^2^S_1/2_ and 3p ^2^P^°^_1/2_–3s ^2^S_1/2_ transitions of Na I at around 589.0 and 589.6 nm, and the 4p ^2^P^°^_3/2–_4s ^2^S_1/2_ and 4p ^2^P^°^_1/2–_4s ^2^S_1/2_ transitions of K I at around 766.5 and 769.9 nm. Importantly, we have not observed other harmful metal pollutants such as Al in the spectra. It is noteworthy that there is no spectral signal observed for Sample S1, indicating the fact that S1 is distilled water. It can be seen that for the drinkable water of Samples S2 and S3, the metal spectral signal intensities are weaker than those of Samples S4–S6, indicating the higher concentration of metal elements in the tap water and lake water.

**Figure 6:**
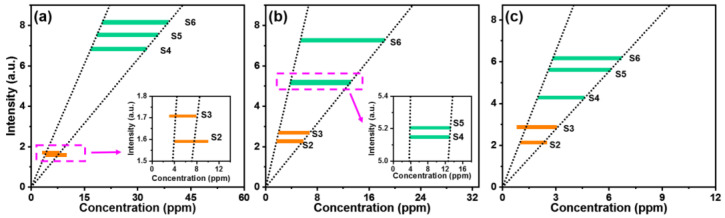
Concentration evaluation of Ca (**a**), Na (**b**), and K (**c**) of Samples S4–S6 based on the concentration data of Samples S2 and S3 given by the manufacturers.

The mineral concentrations of Samples S2 and S3 provided by the manufacturers are listed in Table 1. According to these values, we plot in Figure 6a–c the FIBS intensities of Ca I at 442.7 nm, Na I at 589.0 nm, K I at 766.5 nm as a function of the concentration for Samples S2 and S3, respectively, as indicated by the orange bars. Furthermore, by referencing the mineral concentration ranges of Samples S2 and S3, we fitted the spectral intensities of the Ca, Na, and K elements of Samples S4–S6 and obtained the concentration ranges of these samples, as listed in Table 1. In the fitting procedures, we first determined the concentration boundaries from the concentration ranges of S2 and S3, in which the distance between the two fitting lines in the direction parallel with the x axis is the maximum, but each fitting line crosses with at least one point on both the orange bars, as shown in Figure 6a–c. Based on the boundaries determined by the two fitting lines, we then evaluated from the FIBS intensities the three elemental concentration ranges in Samples S4–S6, as illustrated by the green bars in Figure 6a–c. Clearly, the concentrations of all these three metal elements are higher in Nanhu Park than those in Jingyuetan National Forest Park, showing the ability of this technique in distinguishing the water quality of different environments.

## 4. Summary

In summary, we have experimentally demonstrated that optimizing the laser chirp can significantly improve the sensitivity of the FIBS technique for sensing trace-level metal elements in water. We have also revealed that the FIBS intensity exhibits an asymmetric enhancement along the negative and positive chirps. Based on the ultrafast photoionization model of water in an intense femtosecond laser field, the asymmetric enhancement has been interpreted as the higher MPI rate induced by the higher photon energy in the front part of the negatively chirped pulse, showing the important role of the semiconductor property of water in FIBS. With this understanding, we have realized the improvement of the LOD by one order of magnitude using the chirped FIBS technique. Under the optimal chirp condition, we have performed the FIBS measurements for several water samples including commercial mineral water, tap water, and lake water and estimated the concentrations of Ca, Na, and K elements. Our results show the functionality of the FIBS technique in detecting trace-level metal contents of water, and provide new insights into the filament–water interaction for application of the FIBS technique in monitoring the water quality in a variety of environments.

## Figures and Tables

**Table 1:** Metal element concentrations.

Sample	Ca	Na	K
Concentrations given by the manufacturers			
Nongfu Spring (S2)	4.0~10.0	2.0~6.8	1.0~2.5
Quanyangquan (S3)	3.1~7.9	1.7~5.8	0.8~3.0
Concentrations calculated by the FIBS technique			
Tap water (S4)	18.5~35.7	3.8~13.2	2.6~6.1
Jingyuetan Park (S5)	16.8~32.5	3.8~13.0	2.0~4.6
Nanhu Park (S6)	20.0~38.6	5.4~18.4	2.8~6.7

Unit: ppm.

## Data Availability

Not applicable.
